# Psychosocial and Occupational Factors Associated With Low Back Pain Among Healthcare Professionals: A Systematic Review

**DOI:** 10.7759/cureus.77426

**Published:** 2025-01-14

**Authors:** Maryam A Alammari, Abdulaziz A Alammari, Amal A Alammari, Abdullmajed A Alammari, Nawal A Alammari, Mona Yahya Ibrahim Alabdali

**Affiliations:** 1 Family Medicine, Al-Khobar Health Network, Al-Khobar, SAU; 2 Dental College, King Khalid University, Abha, SAU; 3 Pharmacy, Abha Maternity and Children Hospital, Abha, SAU; 4 Radiology, King Khalid University Hospital, Abha, SAU; 5 Radiology, Aseer Central Hospital, Abha, SAU; 6 Quality, King Khalid University Hospital, Abha, SAU

**Keywords:** health care providers, low back pain, occupational health, occupational risk factors, physical workload

## Abstract

Low back pain (LBP) is a highly prevalent condition among healthcare providers (HCPs) worldwide. The physical demands of their jobs predispose them to risk of LBP and other musculoskeletal disorders (MSDs). LBP is multifactorial and is thus influenced by psychosocial and occupational risk factors. LBP affects the health and productivity of HCPs and needs to be addressed. This review investigated the psychosocial and occupational factors associated with LBP among HCPs. This research adhered to the Preferred Reporting Items for Systematic Reviews and Meta-Analyses (PRISMA). Key medical research databases were searched for relevant studies published between 2016 and 2024. Elaborate inclusion/exclusion criteria were implemented to filter down to 14 full-text studies that were included in this systematic review. The 14 studies pointed toward a varying, high prevalence of LBP among HCPs across different regions. Key risk factors for LBP were high physical workload, poor ergonomics, and psychosocial stressors such as high stress levels, job dissatisfaction, and emotional exhaustion. Shift patterns, workload, and other occupational factors influenced LBP prevalence. High physical workload, poor ergonomics, and repetitive tasks were identified as significant risk factors for LBP. Comprehensive interventions targeting ergonomic training, stress management programs, workplace policy modifications, and targeted strategies for high-risk groups are recommended to address the highly prevalent LBP among HCPs.

## Introduction and background

A primary contributor to years lived with disability (YLDs) is the highly prevalent low back pain (LBP). Research shows that LBP causes significant individual distress globally - people with chronic disabling symptoms also add considerable costs to society through increased healthcare expenses and decreased work productivity [1].

People in varying professional fields like accountants and others who sit at the desk for hours struggle with LBP. Prolonged sitting, poor ergonomics, and work-related stress lead to LBP among white-collar professionals. LBP is an important topic of focus for people across many occupations given its impact on the quality of life (QoL) of affected individuals.

A 2018 study by Cheung et al. estimated that the lifetime prevalence of LBP among healthcare professionals (HCPs) aged between 30 and 49 years was 66.6% [2,3]. Musculoskeletal disorders (MSDs) among hospital staff show varying frequencies across different regions of the world - LBP is the most prevalent and expensive MSD among HCPs in the Americas and European countries where incidences are well-documented [2]. HCPs are more prone to LBP due to their physically demanding roles and ergonomic hazards, often involving lifting patients, prolonged standing, and repetitive movements. Other factors like stress, inadequate sleep, and fatigue during the day are also attributable to LBP prevalence among HCPs [4].

MSDs among healthcare professionals exhibit varying frequencies depending on the specific occupational roles and associated physical demands. For instance, nurses frequently experience MSDs related to patient handling, while radiographers may develop MSDs due to repetitive postures during imaging procedures. A notable statistic in the healthcare profession is that nurses experience the highest incidence of LBP relative to other HCPs [5]. LBP incidences are so high that thousands of nurses worldwide face reduced productivity, require medical care, and may retire early due to LBP [6]. Previous journal articles have pointed out that nurses in intensive care units face disproportionately higher risks of lifelong LBP because of prolonged hours, high workload, and extended patient interaction times [7-9].

Shift patterns, patient conditions, leisure activities, patient weight, and other occupational factors significantly contribute to LBP incidences among HCPs [10,11]. Nurses experience the highest incidence of LBP compared to other HCPs [5]. LBP incidences are so high that thousands of nurses worldwide face reduced productivity, require medical care, and may retire early due to LBP [6].

Studies show that age strongly predicts LBP prevalence - age is positively correlated with LBP incidence among HCPs [12,13,14]. Medical research studies on the interaction between muscle and bone in the elderly found that old age increases the risk of osteoporosis, supporting muscles weaken, and the probability of physical injuries grows due to longer years of work [15,16]. Patients’ physical inabilities and inability to control movement also affect the activity of HCPs.

The interaction between these psychosocial and occupational factors often amplifies the risk of LBP in healthcare professionals, creating a complex web of influences that necessitates a holistic approach to understanding and mitigating these risks [14]. HCPs must give specific attention to and care to such patients due to physical immobilization, which hinders effective care to them and other patients. Frontline health workers also report requiring help handling such patients, sustaining back pains when lifting them, and thus losing working days on their part [17,18,19,20]. This shows the multidimensional manifestations of LBP among HCPs and how it affects their work and personal lives. This systematic review is, therefore, meant to investigate the psychosocial and occupational factors associated with LBP among healthcare professionals.

## Review

Methodology

Literature Search Strategy

This study utilized the guidelines set forth by Preferred Reporting Items for Systematic Reviews and Meta-Analyses (PRISMA), through which five medical databases were searched for relevant scholarly publications published between 2016 and 2024. These databases include PMC, PubMed, Cochrane Library, ScienceDirect, and Google Scholar. We utilized a series of keywords tailored to each database. These keywords included "low back pain, LBP", "healthcare professionals, HCPs", "psychosocial factors", "occupational factors", "nurses", "physicians", "ergonomics", and "workplace stress".

Eligibility, Data Extraction, and Management

We used the Rayyan.ai tool to rigorously assess the retrieved articles for eligibility by comparing them with the predefined inclusion and exclusion criteria all the researchers involved in this study agreed upon.

Inclusion criteria: Studies focusing on LBP, musculoskeletal disorders, and HCPs examining the relationship between psychosocial and occupational factors and LBP. Observational studies included both cohort and cross-sectional designs, with measures of association tailored to each type (prevalence ratios for cross-sectional studies and risk ratios for cohort studies). Additionally, interventional studies were limited to randomized controlled trials (RCTs) and quasi-experimental designs. The primary outcome of interest was a relationship between LBP and medical personnel’s psychological, social, and occupational factors. Inclusion was limited to articles published in English and between 2016 and 2024.

Exclusion criteria: Studies were excluded if they were systematic reviews, meta-analyses, case reports, case series, or qualitative studies. Observational studies without appropriate measures of association (e.g., prevalence ratios or risk ratios) or interventional studies that were not RCTs or quasi-experimental designs were also excluded. Articles not focused on HCPs or those not addressing LBP in relation to psychosocial or occupational factors were omitted. Studies that lacked quantitative measures of association or outcomes related to LBP, were published outside the timeframe of 2016-2024, or were not in English were also excluded.

Two reviewers independently screened all retrieved studies based on the predefined inclusion criteria. We flagged any discrepancies in initial judgments for further discussion.

Quality Assessment

The quality of the included studies was evaluated using the Newcastle-Ottawa Quality Assessment Scale (NOS). This tool assessed the risk of bias across three domains: selection, comparability, and outcome. Studies were classified as having a high, moderate, or low risk of bias based on predefined scoring thresholds: high bias, studies scoring 0-3; moderate bias, studies scoring 4-6; and low bias, studies scoring 7-9. This scoring framework was explicitly defined to enhance transparency and reproducibility.

Statistical Data Analysis

Data were analyzed using Review Manager (RevMan) version 5.4.1 software. The statistical analysis of the systematic review involved evaluating the quality and risk of bias of the included studies. Retrospective studies were assessed using the NOS), where studies were rated on selection, comparability, and outcome. Publication bias was assessed using a funnel plot.

Results

**Figure 1:**
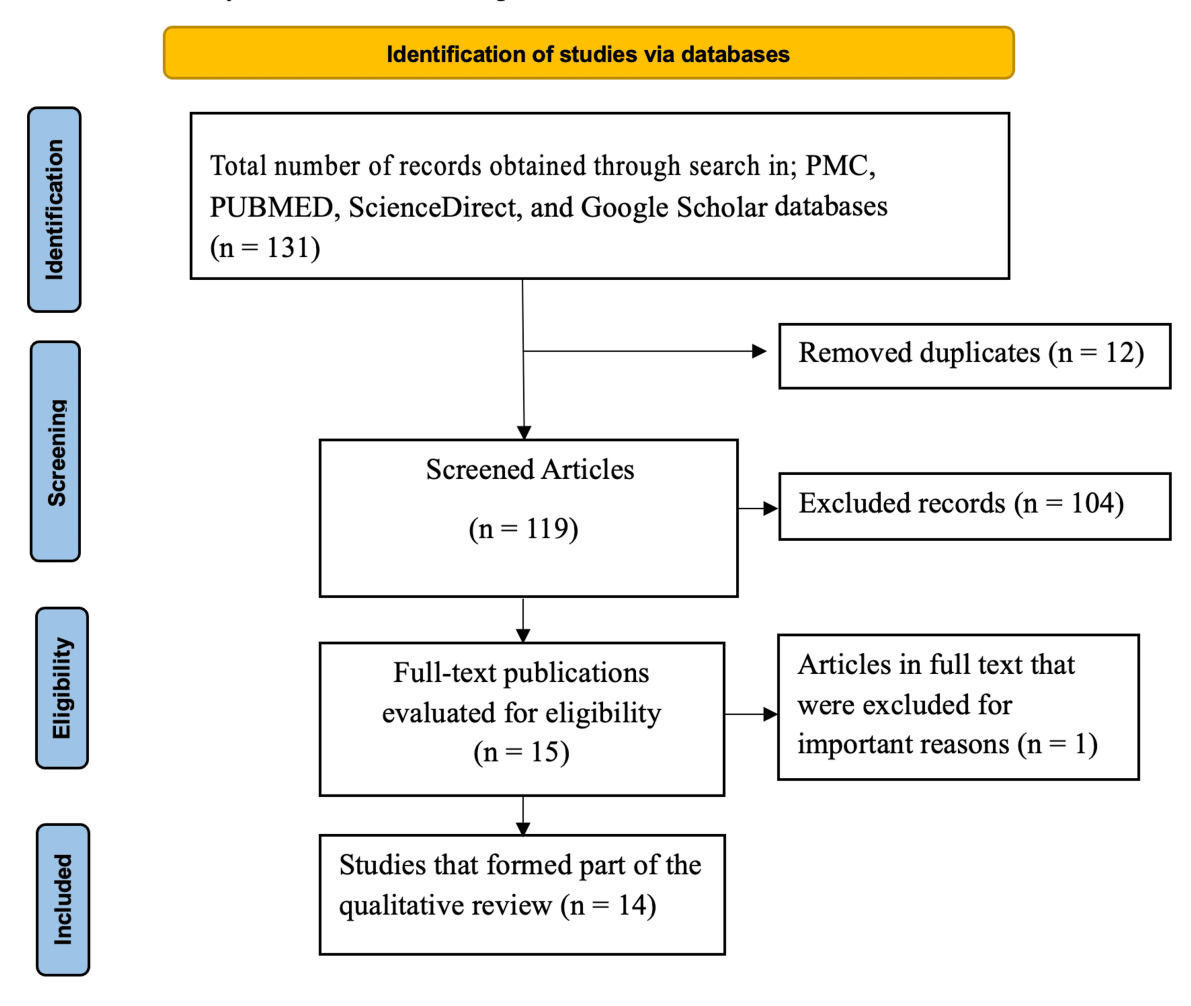
PRISMA flow diagram. PRISMA, Preferred Reporting Items for Systematic Reviews and Meta-Analyses

The PRISMA diagram outlines the systematic process of identifying and including studies in the qualitative review. Initially, 131 records were identified through database searches in PMC, PubMed, ScienceDirect, and Google Scholar. After removing 12 duplicate records, 119 unique articles remained for screening. During the screening phase, 104 articles were excluded based on their titles, abstracts, or lack of relevance to the research question, such as studies not focused on LBP, not involving HCPs, or not meeting language or publication year criteria. This left 15 articles that underwent a full-text review during the eligibility phase. Of these, one article was excluded for not involving HCPs. Thus, 14 studies were deemed eligible and included in the qualitative review, as illustrated in Figure 1.

Study Characteristics

The key attributes of the included studies for review are detailed in Table 1. All the studies included for review were conducted in various regions of the world and were all published in the English language.

**Table 1:** Characteristics of the included studies. MSDs, musculoskeletal disorders; LBP, low back pain; SDs, sleep disorders; PHU, primary health unit; FABs, fear-avoidance beliefs; MSP, musculoskeletal pain; ICU, intensive care unit; BMI, body mass index; MetS, metabolic syndrome; LTCFs, long-term care facilities; RDQ, Roland-Morris Disability Questionnaire

Authors	Study type	Intervention	Sample size	Outcome/Results	Conclusions
Hämmig, 2020 [21]	Cross-Sectional Study	Data were collected on physical workload, stress, and health outcomes.	1,232	Nearly 25% of health professionals reported severe MSDs, and about 14% had severe SDs, more prevalent among nurses than physicians. Key risk factors for MSDs included non-work- and work-related stress, physical effort, and poor posture.	MSDs are mainly due to physical workload and poor posture, while SDs are primarily stress-related. Combining measures to reduce psychological stress and physical strain is necessary.
Fallon et al., 2023 [22]	Cohort Study	Conducted an online survey with 151 radiographers in Ireland to assess LBP prevalence and identify causative factors. Collected demographic data, LBP experiences, activity impacts, and opinions on manual handling and assistive devices.	151	LBP prevalence was 50%, 25% reduced work activity, 43% reduced leisure activities, and 37% sought medical advice due to LBP. Most reported LBP was not due to extracurricular activities.	LBP prevalence among Irish radiographers is high, significantly exceeding the general population rate.
Corrêa et al., 2021 [23]	Observational Study	Surveyed 89 PHU nursing personnel in Pelotas, Brazil, to measure LBP prevalence. Collected sociodemographic, occupational, nutritional, health, and behavioral data. Used Poisson regression to analyze relationships between LBP and various factors.	89	LBP prevalence: 65.2%, with chronic LBP at 22.4% and acute LBP at 53.4%. LBP was linked to obesity and poor self-perceived health, with prevalence ratios of 1.39 and 2.77, respectively.	LBP prevalence among PHU nursing personnel is high, comparable to other nurses. BMI and health perception are significantly associated with LBP.
Ayane et al., 2023 [24]	Cross-Sectional Study	A cross-sectional study of 440 nurses in South-East Ethiopia's Oromia region hospitals. Data were collected via systematic random sampling and analyzed using logistic regressions.	440	42.6% prevalence of LBP, linked to factors like age, gender, and marital status.	Implement preventive measures and ergonomic training to reduce LBP among nurses.
Fujii et al., 2019 [25]	Observational Study	3,066 female nurses from 12 hospitals in Japan were surveyed using a self-reported questionnaire to assess LBP, work-related factors, and FABs about physical activity.	3,066	The four-week and one-year LBP prevalence was 58.7% and 75.9%, respectively. High FABs were significantly associated with chronic disabling LBP (AOR = 1.76, 95% CI [1.21-2.57], *P* = 0.003).	LBP is prevalent among nurses in Japan. Targeting FABs about physical activity could be effective in managing LBP in this population.
Gashawbeza and Ezo, 2022 [26]	Cross-Sectional Study	A cross-sectional study of 470 healthcare providers in Southern Ethiopia's Gamo zone. Data were collected through interviews and anthropometric measurements.	470	LBP prevalence among healthcare providers was 44.2%. Significant risk factors included systemic illness, posture, night work, lifting heavy weights, and job satisfaction. The belief that night work aggravated LBP was also notable.	Approximately 40% of healthcare providers in the Gamo zone experience LBP. Ergonomic equipment, proper lifting techniques, and alternating posture may help reduce this burden.
Jradi et al., 2020 [27]	Cross-Sectional Study	Cross-sectional survey of 427 nurses across 16 hospitals in Riyadh, Saudi Arabia. Assessed psychosocial and occupational factors linked to LBP. Found an 80% prevalence of LBP.	427	LBP prevalence was 80%. Key factors: frequent lifting, work-related stress, lack of job satisfaction, work-related problems, and financial problems.	LBP prevalence is high among healthcare workers. Address: ergonomic and psychosocial factors, stress reduction, counseling, and policies to improve job satisfaction.
Freimann, et al., 2016 [28]	Cross-Sectional Study	Survey of 409 nurses at Tartu University Hospital. Assessed the prevalence of MSP and its associations with work-related psychosocial factors and mental health problems. Found that 70% of nurses experienced MSP in the past year.	409	Lower back (57%) and neck (56%) were the most affected areas. 70% experienced MSP in the past year, with 64% experiencing it in the past month. Mental health issues, particularly somatic stress symptoms, were also linked to MSP.	Work-related psychosocial factors and mental health issues like somatic stress symptoms impact the occurrence of MSP among university hospital nurses. Addressing these factors is crucial for reducing MSP.
Pellissier et al., 2023 [29]	Observational Study	Surveyed 604 French physiotherapists to study the prevalence of work-related LBP and associated risk factors. Compared practice patterns, LBP prevalence, and exposure to biomechanical, psychosocial, and organizational risks.	604	40.4% reported nonspecific, work-related LBP in the past 12 months. LBP prevalence was higher in geriatrics and lower in sports medicine. Variations in exposure to risk factors were noted.	The risk of non-specific LBP among French physiotherapists varies by practice mode. This study provides a basis for tailored research on the most exposed practices.
Vinstrup et al., 2020 [30]	Cohort Study	A prospective cohort study of 1,944 Danish healthcare workers from 19 hospitals. Used Cohen's Perceived Stress Scale to assess baseline stress levels and their association with LBP at a 1-year follow-up.	1,944	Moderate and high stress levels at baseline increased the odds of LBP at one-year follow-up - odds ratios of 1.39 and 1.99, respectively. Similar results were found for female nurses, with high stress increasing LBP odds for those without baseline LBP.	Psychological stress significantly raises the odds of LBP among healthcare workers. Strategies to prevent musculoskeletal disorders should focus on reducing work-related psychosocial stressors.
Wang et al., 2022 [31]	Observational Study	Conducted an online survey of 356 ICU personnel at West China Hospital of Sichuan University. Assessed the prevalence and risk factors of pain using sociodemographic, ergonomic, and psychological data.	356	Pain prevalence: 72.2% for nurses, 64.4% for doctors, and 52.9% for workers. Lower back pain was most common among nurses (65.9%) and workers (47.1%); neck pain was prevalent among doctors (49.1%). Key risk factors: neck bending, psychological fatigue, low self-perceived health, female sex, and high BMI.	ICU personnel have a high prevalence of pain, influenced by psychosocial and ergonomic factors. Implementing disease prevention and health promotion measures is essential to protect their health.
Tsuboi et al., 2018 [32]	Cross-Sectional Study	A cross-sectional study of 316 care workers to investigate the association between MetS and disability due to LBP. Used the Roland-Morris Disability Questionnaire and self-reported questionnaires to assess LBP.	316	16.5% were diagnosed with MetS. MetS was associated with higher RDQ scores for disability due to LBP after adjusting for various factors, with an adjusted prevalence ratio of 1.57 (95% CI: 1.17-2.11).	MetS is independently linked to LBP disability among care workers. Multidisciplinary interventions considering MetS could effectively reduce LBP-related disability.
Yang et al., 2021 [33]	Cross-Sectional Study	Involved 308 nursing aides from LTCFs in Taiwan. Used demographic surveys, job content questionnaires, and musculoskeletal questionnaires to assess the association of work-related psychological factors with MSDs.	308	Lower job control and higher psychological job demands, low social support, were significantly associated with more severe MSDs among nursing aides. Key factors: nationality, age, exercise habits, chronic diseases, worksite conditions, lack of rest time, lack of assistive devices, and low coworker support. These factors explained 42.1% of MSD prevalence.	Work-related psychological factors significantly impact MSDs among nursing aides in LTCFs. Enhancing coworker support and reducing psychological job demands are recommended.
Zhang et al., 2019 [34]	Cross-Sectional Study	A cross-sectional study of 1,560 ambulance workers in Shandong, China. Used questionnaires to assess LBP, ergonomic factors, and stress.	1,560	One-year prevalence of LBP lasting at least three months was 21.1%, 15.8%, and 12.3% among respondents. Factors for chronic LBP in ambulance nurses: frequent trunk bending, heavy lifting, long-shift work, high psychological fatigue, high job demand, low job control, low supervisor support, older age, gender, and obesity.	LBP is more prevalent among ambulance nurses compared to doctors and drivers. Psychosocial and ergonomic factors play significant roles, indicating the need for comprehensive measures to control LBP.

Assessment of Item Risk of Bias of the Retrospective Studies

The quality of the retrospective studies was evaluated using the NOS (Table 2). Of the 14 studies, 5 were found to be of high quality with a low risk of bias [22,24,25,31,33]. On the other hand, of the 14, 6 had a moderate risk of bias [21,23,26,29,30,32] and 3 had a high risk of bias [27,28,34]. The biases were attributed to various types of selection, comparability, and outcomes, as illustrated in Table 2.

**Table 2:** Newcastle-Ottawa Quality Assessment Scale (NOS) for the included studies. For every numbered item in the selection and outcome categories, a study received up to one star (*) for assessment of each item. However, comparability received a rating of up to two stars based on the assessment results (**). For selection and outcome assessment, one star (*) shows a low risk of bias for each item assessed. On the other hand, for comparability assessment, two stars (**) show a low risk of bias, while (-) shows a high risk of bias for each item assessment.

Author	Selection				Comparability	Outcome		
	Representativeness of the exposed cohort	Selection of the non-exposed cohort	Ascertainment of exposure	Demonstration that outcome of interest was not present at the start of the study	Comparability of cohorts on the basis of the design or analysis	Assessment of outcome	Was follow-up long enough for outcomes to occur	Adequacy of follow-up of cohorts
Hämmig, 2020 [21]	*	-	*	*	**	*	*	*
Fallon et al., 2023 [22]	*	*	*	*	**	*	*	*
Corrêa et al., 2021 [23]	*	*	*	*	**	*	*	-
Ayane et al., 2023 [24]	*	*	*	*	**	*	*	*
Fujii et al., 2019 [25]	*	*	*	*	**	*	*	*
Gashawbeza and Ezo, 2022 [26]	*	*	*	*	*	*	*	*
Jradi et al., 2020 [27]	*	*	*	*	**	*	*	*
Freimann et al., 2016 [28]	*	*	*	*	*	*	*	-
Pellissier, 2023 [29]	*	*	*	*	*	*	*	*
Vinstrup et al., 2020 [30]	*	-	*	*	**	*	*	*
Wang et al., 2022 [31]	*	*	*	*	**	*	*	*
Tsuboi et al., 2018 [32]	*	*	*	*	*	*	*	*
Yang et al., 2021 [33]	*	*	*	*	**	*	*	*
Zhang et al., 2019 [34]	*	-	*	*	*	*	-	*

Assessment of Publication Bias of the Included Studies

**Figure 2:**
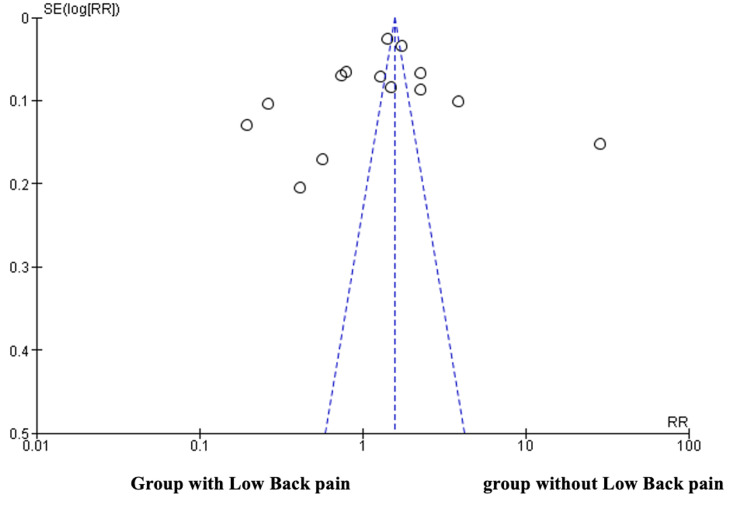
Funnel plot depicting publication bias. The x-axis represents the standard error, and the y-axis represents the effect size. SE, standard error; RR, relative risk.

An asymmetrical funnel is depicted in Figure 2, with more studies aligned to the left than to the right. Nevertheless, there are more outliers aligned on the left side of the funnel than on the right side. On that basis, the results suggest that there are chances of having publication bias.

Discussion

This systematic review aimed to provide valuable insights into the interplay between psychosocial and occupational factors and LBP among HCPs. A total of 14 studies were included, offering a global perspective on the prevalence of LBP and its associated risk factors. The findings highlight significant variations in prevalence rates and risk factors for LBP across regions and professions, influenced by both ergonomic and psychosocial factors.

The studies underscored the role of the work environment, including shift patterns and workload, in influencing LBP. Freimann et al. found that high work demands, low workplace justice, influence on work organization, and role conflicts were the key factors causing the highly prevalent MSP (70%) among the 409 nurses at Tartu University Hospital [28]. However, Tsuboi et al. took a relatively different approach by assessing the link between metabolic syndrome (MetS) and disability due to LBP [32]. A higher Roland-Morris Disability (RDQ) result indicated a positive correlation between chronic LBP and disability. MetS was independently linked to LBP disability among HCPs.

The studies consistently identified high physical workload, repetitive movements, and poor ergonomics as primary contributors to LBP. HCPs frequently perform tasks such as lifting patients, working in awkward positions, and standing for prolonged periods, which place significant strain on the musculoskeletal system. Zhang et al. found that ambulance workers were more likely to develop LBP due to psychosocial and occupational factors associated with their work [34] - frequent trunk bending, heavy lifting, poor job satisfaction, high mental and physical fatigue, high job demand, low job control, and low supervisor support. Fujii et al. noted a one-year prevalence of 59% among Japanese nurses, correlating it with high physical demands and insufficient ergonomic interventions [25].

Psychosocial factors, including stress, burnout, and low job satisfaction, were equally impactful. Hämmig surveyed 1,232 health professionals in Swiss hospitals and clinics to study musculoskeletal and sleep disorders [21]. Nearly 25% of respondents reported severe LBP. This study highlights the substantial impact of LBP on healthcare workers in Swiss hospitals, correlating high physical workload and psychosocial stress as primary factors. Similarly, Jradi et al. accessed psychosocial and occupational factors that caused LBP across 16 hospitals in Riyadh, Saudi Arabia [27]. A univariant analysis indicated similar psychosocial and occupational LBP risk factors like work-related stress and a lack of job satisfaction. Vinstrup et al. also linked stress levels as a predisposing factor to LBP among the 1,944 Danish HCPs [30].

Prevalence rates varied across regions due to psychological and physical risk factors. Corrêa et al. [23] reported a 65.2% prevalence among Brazilian public health unit nurses, with chronic LBP affecting 22.5%. Similarly, Ayane et al. also emphasized the exacerbation of LBP due to high stress levels and emotional exhaustion among HCPs in South Africa [24]. Similarly, Fallon et al. [22] observed a 50% prevalence among Irish radiographers, underscoring the physical and ergonomic challenges specific to this profession. The differences may be attributed to systemic and policy-related factors, such as the availability of ergonomic training, workplace safety regulations, and access to healthcare. Cultural perceptions of pain and reporting practices could also play a role, as they may influence how LBP is identified and addressed in different regions.

Limitations

This review has some limitations. First, only studies published in English were included, which may introduce language bias and exclude relevant data from non-English sources. This exclusion is particularly significant given the global scope of LBP studies and warrants future efforts to incorporate multilingual research. Additionally, there was considerable heterogeneity in the included studies regarding sample populations, professional roles, and definitions of LBP. For instance, some studies lacked clear definitions of LBP severity or duration, which could affect the consistency of findings. To mitigate publication bias, funnel plots or Egger’s test could be utilized thoroughly to assess LBP prevalence and causes, in future systematic reviews. Moreover, incorporating non-English studies and engaging a diverse group of reviewers to assess eligibility criteria could help reduce potential biases.

## Conclusions

There was a significant prevalence of LBP among HCPs globally. High physical workload, poor ergonomics, and repetitive movements were common contributors to chronic LBP, particularly in roles requiring patient handling and prolonged standing. Psychosocial stressors, including high stress levels, job dissatisfaction, and emotional exhaustion, further exacerbated LBP among HCPs, particularly in environments with demanding workloads and inadequate support. Shift patterns, workload, and the type of healthcare practice were identified as the main work environmental factors that played a crucial role in influencing LBP prevalence. Comprehensive interventions targeting these areas are essential to improve the well-being of HCPs and enhance overall productivity and patient care in healthcare settings. Most of the studies recommended ergonomic training, stress management programs, workplace policy modifications, and targeted strategies for high-risk groups as the key strategies to mitigate the risk of LBP among HCPs.
